# Partial Oculomotor Nerve Palsy Revealing PDCD10-Related Familial Cerebral Cavernous Malformation in a Child

**DOI:** 10.7759/cureus.111335

**Published:** 2026-06-22

**Authors:** Yoshimi Murakami, Takanobu Inoue, Shun Ishikura, Ryo Fujimoto, Hiromi Aoyama

**Affiliations:** 1 Division of Child Neurology, Chiba Children's Hospital, Chiba, JPN; 2 Center for Medical Genomics, Chiba Children's Hospital, Chiba, JPN

**Keywords:** brainstem lesion, familial cerebral cavernous malformation, infant, oculomotor nerve palsy, pdcd10 variant

## Abstract

Familial cerebral cavernous malformation (FCCM) is a hereditary vascular disorder associated with pathogenic variants of *KRIT1 (CCM1), CCM2*, and *PDCD10 (CCM3)*. Among these, *PDCD10*-related FCCM is considered the most severe subtype and is characterized by early onset, multiple lesions, frequent brainstem involvement, and an increased risk of intracranial hemorrhage. Brainstem symptoms may be the initial manifestations. However, isolated oculomotor nerve palsy in childhood is rare.

We report the case of a 17-month-old girl with normal developmental milestones who developed mild right ptosis, strabismus, and impaired adduction and upward gaze of the right eye at nine months of age. There was no family history of seizures or intracranial hemorrhage. Computed tomography of the head revealed multiple intracranial calcifications. Susceptibility-weighted angiography (SWAN) magnetic resonance imaging (MRI) revealed multiple cerebral cavernous malformations (CCMs), and follow-up MRI one year later demonstrated new lesions, including pontine involvement. Fast imaging employing steady-state acquisition (FIESTA) revealed hemorrhagic changes adjacent to the right oculomotor nerve within the interpeduncular cistern, suggesting that the partial oculomotor nerve palsy was associated with focal hemorrhagic involvement. Genetic analysis revealed the presence of a novel *PDCD10* variant.

This case highlights that *PDCD10*-related FCCM may initially present with isolated cranial neuropathy during childhood and subsequently show rapid radiological progression. Careful longitudinal follow-up is required because of the risk of future neurological deterioration.

## Introduction

Cerebral cavernous malformations (CCMs) are venous malformations composed of dilated capillary-like vessels without intervening brain parenchyma that occur sporadically or in a familial form [1].

These vascular channels are lined by a single layer of endothelial cells and lack mature vessel wall structures. Familial cerebral cavernous malformation (FCCM) is an autosomal dominant disorder associated with pathogenic variants of *KRIT1 (CCM1), CCM2*, and *PDCD10 (CCM3)* [1]. These genes are involved in the maintenance of endothelial barrier integrity, and their dysfunction contributes to increased vascular permeability and intracranial hemorrhage [2]. Patients with FCCM characteristically develop multiple lesions and may present with seizures, headaches, focal neurological deficits, and intracranial hemorrhage [1].

Among the FCCM-associated genes, *PDCD10*-related FCCM is considered the most severe subtype and is characterized by early onset, multiple lesions, rapid lesion progression, frequent brainstem involvement, and an increased risk of intracranial hemorrhage [3,4]. In a genotype-phenotype cohort study, 26% of patients developed symptoms before 10 years of age, with cerebral hemorrhage being a frequent initial manifestation among these early-onset cases [5]. Patients may develop early intracranial hemorrhage and severe neurological symptoms that require careful long-term follow-up [4]. Therefore, the identification of a *PDCD10* variant provides important clinical information regarding disease severity and long-term prognosis.

FCCM initially presenting with oculomotor nerve palsy is relatively uncommon, and no previous reports have described pediatric *PDCD10*-related FCCM presenting with isolated oculomotor nerve palsy. Cavernous malformations adjacent to the brainstem or oculomotor nerve may cause ptosis, strabismus, and ocular movement disturbances via focal hemorrhage or neural compression [6].

Here, we report the case of a 17-month-old girl with *PDCD10*-related FCCM who presented with partial oculomotor nerve palsy during childhood and demonstrated rapid radiological progression on serial magnetic resonance imaging (MRI) scans.

## Case presentation

A previously healthy 17-month-old girl with normal developmental milestones was referred to the ophthalmology department with mild right ptosis, strabismus, and limited ocular movements that had appeared at nine months of age. There was no known family history of intracranial hemorrhage or seizures. Computed tomography (CT) of the head demonstrated multiple calcified lesions in the cerebral white matter (Figure 1), and the patient was referred to our department for further evaluation.

**Figure 1:**
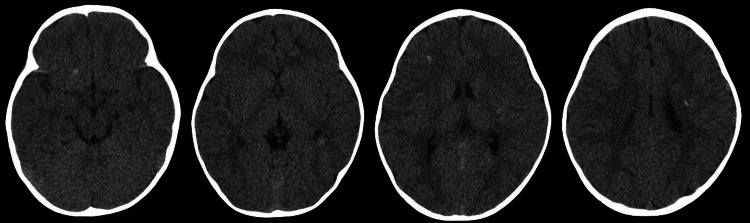
CT of the patient’s head Axial head CT images demonstrating multiple calcified lesions in the cerebral white matter. CT, computed tomography

Neurological examination revealed mild right ptosis, exotropia, impaired adduction, and impaired upward gaze of the right eye. No other focal neurological deficits were identified, and the patient’s general condition was good. Blood tests revealed no significant abnormalities. Brain MRI with susceptibility-weighted angiography (SWAN) demonstrated multiple hypointense lesions throughout the brain parenchyma, consistent with multiple CCMs (Figure 2).

**Figure 2:**
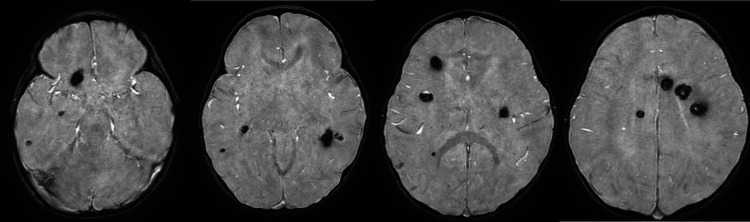
Brain MRI with SWAN SWAN images demonstrating multiple hypointense lesions throughout the brain parenchyma, consistent with multiple CCMs. SWAN, susceptibility-weighted angiography; CCM, cerebral cavernous malformation; MRI, magnetic resonance imaging

Furthermore, fast imaging employing steady-state acquisition (FIESTA) revealed hemorrhagic changes adjacent to the right oculomotor nerve within the interpeduncular cistern, suggesting that the partial oculomotor nerve palsy was associated with a focal hematoma and neural compression (Figure 3).

**Figure 3:**
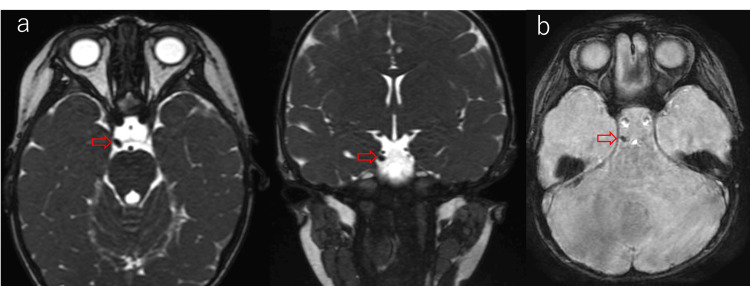
FIESTA and SWAN imaging of the right oculomotor nerve (a) FIESTA image showing hemorrhagic changes adjacent to the right oculomotor nerve within the interpeduncular cistern (red arrow), suggesting a focal hematoma and neural compression associated with partial oculomotor nerve palsy. (b) SWAN image demonstrating a hypointense lesion adjacent to the right oculomotor nerve (red arrow). FIESTA, fast imaging employing steady-state acquisition; SWAV, susceptibility-weighted angiography

Because of the presence of multiple lesions, FCCM was suspected. We performed targeted next-generation sequencing of three CCM-causative genes: *KRIT1 (CCM1), CCM2,* and *PDCD10 (CCM3)*. We identified a novel heterozygous pathogenic variant in *PDCD10*, NM_145860.2.506dup (p.Tyr170ValfsTer7). The identified *PDCD10* variant, NM_145860.2:c.506dup (p.Tyr170ValfsTer7), was not present in the gnomAD population database and has not been reported in ClinVar or HGMD. Therefore, the variant was considered novel. This frameshift variant is predicted to result in premature protein truncation and loss of function. Because loss of function is an established disease mechanism for *PDCD10*-related FCCM, the variant was classified as pathogenic. No parental analyses were performed.

Follow-up MRI performed one year later demonstrated enlargement of the preexisting lesions and the appearance of multiple new lesions, including lesions within the pons (Figure 4), suggesting rapid radiological progression.

**Figure 4:**
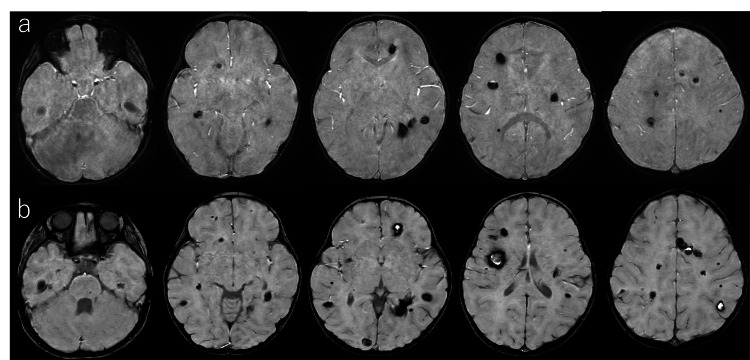
Follow-up SWAN and SWI MRI demonstrating rapid radiological progression a. SWAN image. b. SWI image obtained one year later, demonstrating enlargement of preexisting lesions and the appearance of multiple newly developed lesions, including pontine lesions. SWAN, susceptibility-weighted angiography; SWI, susceptibility-weighted imaging; MRI, magnetic resonance imaging

During 18 months of follow-up, ptosis, exotropia, and ocular movement limitation remained stable, without significant improvement or deterioration. No additional cranial neuropathy, seizures, or developmental regression was observed. Additional spinal MRI revealed no abnormalities. As no major hemorrhage occurred and the neurological symptoms remained stable, there was no indication for surgical intervention. Therefore, the patient was managed conservatively with serial neurological and radiological follow-up.

Written informed consent was obtained from the patient’s legal guardians for participation in this study and for the publication of clinical data and images.

## Discussion

Herein, we report a rare pediatric case of *PDCD10*-related FCCM presenting with partial oculomotor nerve palsy as the initial manifestation.

*PDCD10*-related FCCM is considered the most aggressive subtype and is characterized by a younger age at onset, greater lesion burden, rapid lesion progression, frequent brainstem involvement, and an increased risk of intracranial hemorrhage compared with other FCCM subtypes [3,4]. Previous studies have suggested that *PDCD10* dysfunction impairs endothelial barrier integrity by disrupting endothelial tight junctions and causing abnormalities in the actin cytoskeleton, resulting in vascular instability, hemorrhagic tendencies, and lesion multiplicity [2].

Our patient showed multiple calcified lesions on CT and numerous hypointense lesions on SWAN, consistent with multiple CCMs [7]. In addition, serial MRI demonstrated the rapid development of new lesions, including pontine lesions, within one year. This aggressive radiological progression was considered compatible with the known phenotype of *PDCD10*-related FCCM [3,4]. While previous *PDCD10* cohorts have emphasized early symptomatic hemorrhage, our patient presented with isolated partial oculomotor nerve palsy without major intracranial hemorrhage. This presentation expands the recognized clinical spectrum of *PDCD10*-related FCCM and suggests that focal cranial neuropathy may be an early manifestation of the disease.

Oculomotor nerve palsy is an uncommon presentation of FCCM, particularly in children. In the present case, FIESTA imaging demonstrated hemorrhagic changes adjacent to the right oculomotor nerve within the interpeduncular cistern, suggesting focal hematoma and neural compression as the mechanisms underlying the partial oculomotor nerve palsy. The localized nature of the lesion may explain the selective involvement of oculomotor nerve function, resulting in ptosis, exotropia, and impaired ocular movements without complete third nerve palsy. No aneurysm, tumor, or inflammatory lesion was identified on neuroimaging, making other common causes of pediatric oculomotor nerve palsy unlikely. Therefore, brainstem or cranial nerve involvement may represent an important early manifestation of *PDCD10*-related FCCM even in the absence of seizures or major intracranial hemorrhage [4]. Clinicians should also be aware that acquired oculomotor nerve palsy may be a neurological manifestation of CCM.

This case highlights the clinical importance of genetic testing in pediatric patients with multiple cavernous malformations. Even in the absence of a family history, identification of a *PDCD10* variant may provide useful information regarding disease severity, hemorrhagic risk, and long-term prognosis [8,9]. Because FCCM is inherited in an autosomal dominant manner, genetic counseling and cascade testing of at-risk family members should be considered. Although parental testing has not yet been performed in the present case, evaluation of first-degree relatives may help clarify inheritance patterns and guide long-term surveillance. During 18 months of follow-up, ptosis, exotropia, and ocular movement limitation remained clinically stable despite radiological progression. Conservative management was selected because no major hemorrhage occurred during follow-up, and the neurological symptoms remained stable. We currently perform annual MRI surveillance when the patient remains clinically stable. Surgical intervention would be reconsidered in the event of symptomatic hemorrhage, depending on the lesion location and surgical accessibility. Careful longitudinal neuroimaging and neurological follow-up are required because rapid radiological progression may precede clinical deterioration.

Further accumulation of pediatric cases is necessary to clarify the clinical spectrum and natural history of *PDCD10*-related FCCM presenting with cranial neuropathy during childhood. A limitation of this report is that parental genetic testing was not performed; therefore, segregation analysis could not be performed.

## Conclusions

Here, we report a rare pediatric case of *PDCD10*-related FCCM presenting with partial oculomotor nerve palsy. *PDCD10*-related FCCM carries a high risk of intracranial hemorrhage and may show rapid radiological progression with brainstem lesions, necessitating careful follow-up. Genetic analyses may be useful for assessing disease severity and long-term prognosis. In addition, clinicians should be aware that acquired oculomotor nerve palsy may be a neurological manifestation of CCM.
